# Evidence of Incipient Forest Transition in Southern Mexico

**DOI:** 10.1371/journal.pone.0042309

**Published:** 2012-08-08

**Authors:** Raúl Abel Vaca, Duncan John Golicher, Luis Cayuela, Jenny Hewson, Marc Steininger

**Affiliations:** 1 División de la Conservación de la Biodiversidad, Departamento de Ecología y Sistemática Terrestre, El Colegio de la Frontera Sur, San Cristóbal de Las Casas, Chiapas, México; 2 Centre for Conservation Ecology and Environmental Change, School of Conservation Sciences, Bournemouth University, Fern Barrow, Poole, Dorset, United Kingdom; 3 Área de Biodiversidad y Conservación, Universidad Rey Juan Carlos, Móstoles, Madrid, España; 4 Conservation International, Arlington, Virginia, United States of America; University of Western Australia, Australia

## Abstract

Case studies of land use change have suggested that deforestation across Southern Mexico is accelerating. However, forest transition theory predicts that trajectories of change can be modified by economic factors, leading to spatial and temporal heterogeneity in rates of change that may take the form of the Environmental Kuznets Curve (EKC). This study aimed to assess the evidence regarding potential forest transition in Southern Mexico by classifying regional forest cover change using Landsat imagery from 1990 through to 2006. Patterns of forest cover change were found to be complex and non-linear. When rates of forest loss were averaged over 342 municipalities using mixed-effects modelling the results showed a significant (p<0.001) overall reduction of the mean rate of forest loss from 0.85% per year in the 1990–2000 period to 0.67% in the 2000–2006 period. The overall regional annual rate of deforestation has fallen from 0.33% to 0.28% from the 1990s to 2000s. A high proportion of the spatial variability in forest cover change cannot be explained statistically. However analysis using spline based general additive models detected underlying relationships between forest cover and income or population density of a form consistent with the EKC. The incipient forest transition has not, as yet, resulted in widespread reforestation. Forest recovery remains below 0.20% per year. Reforestation is mostly the result of passive processes associated with reductions in the intensity of land use. Deforestation continues to occur at high rates in some focal areas. A transition could be accelerated if there were a broader recognition among policy makers that the regional rate of forest loss has now begun to fall. The changing trajectory provides an opportunity to actively restore forest cover through stimulating afforestation and stimulating more sustainable land use practices. The results have clear implications for policy aimed at carbon sequestration through reducing deforestation and enhancing forest growth.

## Introduction

The extensive and unrestrained deforestation that affected many tropical regions in the second half of the twentieth century has been extensively documented. The apparent inevitability of continued tropical forest loss and environmental degradation has caused widespread alarm [1]–[3]. Many studies of tropical deforestation reported linear rates of deforestation with little indication that the process could be halted or reversed [4]. However recent studies have provided evidence that tropical regions have begun to show signs of recovery in overall forest cover [5]–[10]. The forest transition model [11], [12] proposes that the changes in the rural economy that are associated with development lead to a more or less predictable reduction in deforestation rates and an eventual increase in afforestation.

The general model underlying forest transition theory is known as the Environmental Kuznets Curve (EKC) [13]. Kuznets postulated that as the human population expands and the economy develops, the comparative importance of direct exploitation of natural resources falls. As a smaller proportion of the total population relies on natural resources for their livelihoods, opportunities arise for restoration of previously over exploited resources. The EKC follows a U-shaped, or a reverse J-shaped form in its incipient phase. Placed in the context of land use change, the EKC predicts a decline in forest cover at an increasingly rapid rate during the first phase of development followed by a gradual stabilization. In the final phase of the transition there is a tendency towards recovery of forest cover, leading to an up turn in the curve [13], [14]. The original empirical evidence in support of this process was drawn from observations in developed nations [11]. Recent studies in developing countries have suggested that some of the initial expectations of forest transition theory were probably overly simplistic [13], [15]. In developing nations a complex and interacting set of demographic, social, ecological, economic and political drivers determine the way land use transitions occur [16]. Tropical forest transitions may be difficult to detect in their initial phase, since case studies often focus on areas where ongoing deforestation is causing particular concern, overlooking neighboring areas that may have entered the stable phase of the transition [17].

There are at least two broad types of processes that are known to lead to forest transitions in tropical countries. 1) The passive transition. In this case farmers become aware of the existence of comparatively better paid off farm jobs and migrate. The opportunity to leave the land may have arisen as a result of economic growth in the country or region in which deforestation has taken place, or in another, usually neighboring, region or country. The result is land abandonment and the possible regeneration of forests on abandoned fields. In some cases ecological degradation and lack of propagules may prevent any forest regeneration taking place. 2) The active transition. In this situation a growing scarcity of forest products or a desire to directly enhance ecological services encourages governments and landowners to plant trees on land that was previously used for agriculture or pasture. Afforestation becomes profitable due to either direct returns or through subsidies made possible by economic growth. Both forms of forest transition are stimulated by economic development in urban areas rather than an increase in rural prosperity.

A forest transition may not enhance biodiversity in the short term due to the low biological value of secondary forests and plantations compared to intact native forest. Degradation of forest quality may also continue to occur in some areas even while forest cover is extending. However areas undergoing transition are likely to sequester more carbon and provide other ecological services such as soil and water conservation. This has important implications for both conservation planning and for the rural economy in Mexico [6]. Recent research on relatively small scale case study areas has documented the occurrence of these different forms of forest transitions [6], [18], [19]. Nevertheless the prevailing paradigm for conservation and natural resource management within the region remains the long established view that deforestation continues to accelerate. The contradictory interpretations of patterns of land use change [6], [18], [20], [21] suggest that any transition in Southern Mexico remains in an incipient phase that would be difficult to detect without large, systematic studies that place cover change dynamics within a regional perspective. One of the few previous efforts in this direction has been that of Rudel [22], who used a method for collating and analysing data from multiple local case studies. Some published literature on forest cover dynamics in Southern Mexico supports the forest transition model [20].

This study aims to rigorously test the evidence for an incipient forest transition in Southern Mexico through a detailed analysis that disaggregates cover change dynamics and analyses the results within the regional context. The spatio-temporal analysis aimed to appropriately contextualize change dynamics, and provide the necessary framework for integrating theory and context specific information [17], [22].

We had four specific objectives:

1. To test the EKC hypothesis directly by analysing the temporal change of deforestation rates over the last 16 years.
2. To test the EKC hypothesis indirectly using space for time substitution to investigate the form of relationships between forest cover and key socio-economic variables.
3. To quantify subregional variability in change trajectories and produce estimates of rates of change by disaggregating results at scales that are appropriate for guiding policy making.
4. To investigate the nature of the process underlying any forest transition though revision of case study based literature.

In order to address these aims we mapped the spatial distribution and quantified the extent of forest cover changes in Southern Mexico, during the periods 1990–2000 and 2000–2006 by classifying Landsat imagery. The technique has been shown to be especially useful for studying change in terrestrial vegetation at regional scale [23], [24].

## Methods

### Study Area

**Figure 1:**
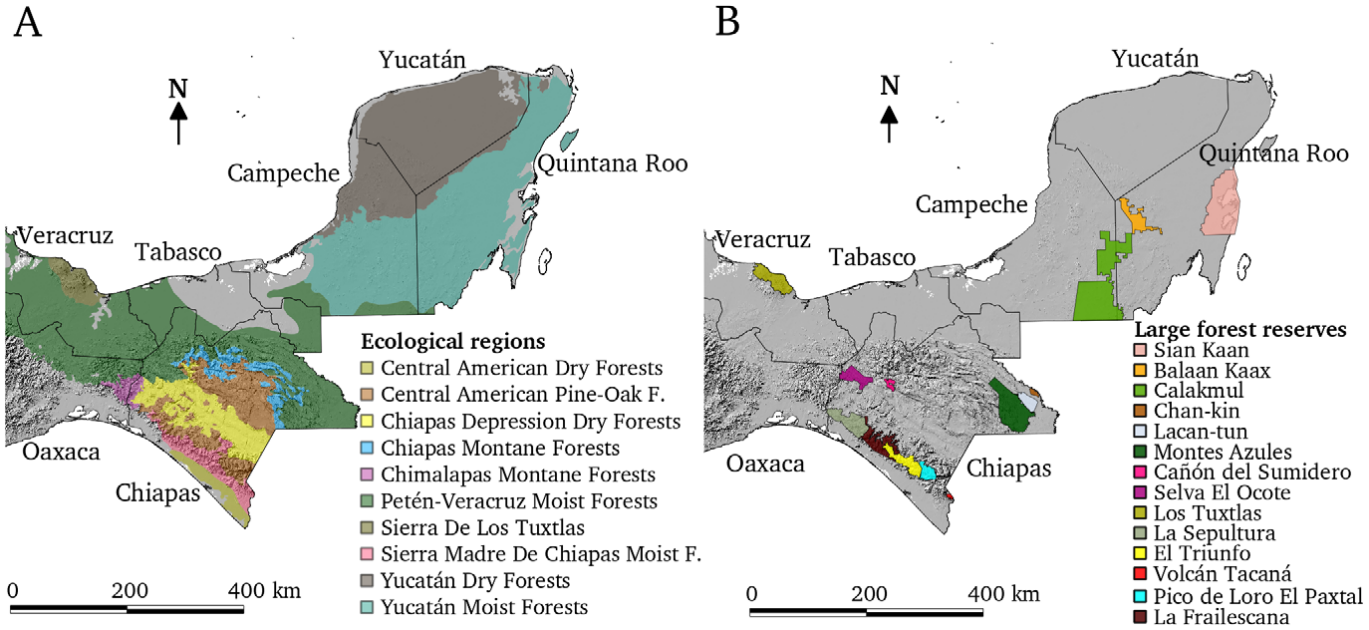
Study area. A) Ecological regions of Southern Mexico as proposed by Olson et al. [37]: gray areas consist in areas excluded from analysis. B) Large forest reserves.

The study area includes the states of Chiapas, Tabasco, Campeche, Quintana Roo, Yucatán, and part of Oaxaca and Veracruz (**Figure 1**). It extends over 287,397 km^2^, including very diverse climatic and physiographic conditions. The physiography of the study area includes mountainous systems, coastal plains and hills, plateaus, valleys and high plains. The main climatic types are: tropical humid, tropical sub-humid and temperate humid, with numerous subtle variations [25].

In the absence of anthropogenic intervention most of Southern Mexico would be covered by forests of some type [26]. Natural differences between forest types are determined by variability in climate and soil type. Elevation largely determines transitions between temperate and tropical forests, while variability in rainfall determines transitions between moist and dry tropical forest [26].

### Satellite Imagery

We used orthorectified Landsat images from NASA’s Geocover project [24], with dates ca. 1990 (1987–1990), ca. 2000 (1999–2001), and ca. 2006 (2005–2007). Images were acquired during the dry season between February and May, or during the beginning of this season between October and February. For the period 1987–1990 we used Landsat Thematic Mapper (TM) data, whereas for the period 1999–2001 and 2005–2007, we used Landsat Enhanced Thematic Mapper Plus (ETM+) data. Missing areas due to sensor malfunctioning were filled using the values from the previous imagery.

### Forest Change Classification

Our analyses were conducted at a 28.5 m spatial resolution. The method used was previously described by Christie et al. [27], Harper et al. [28] and Leimgruber et al. [29], among others. It was specifically designed for cover change analysis.

The cited locational accuracy of TM and ETM+ Landsat data is 50 m [24]. To minimise false change caused by locational inconsistency between dates to less than one pixel width (*i.e.* between 15 and 28.5 m), all dates of Landsat imagery were co-registered.

Data were classified together into single multi-date images (*i.e.* 1990–2000 and 2000–2006 composites). Rather than classifying single-date images individually and then combining them to derive change estimates, classification of multi-date images reduces false-change errors caused by differences between image dates in vegetation phenology, illumination conditions and atmospheric interference. In addition, multi-date composites permit visualization of both image dates simultaneously to determine where change is occurring, therefore it is possible to classify forest cover and forest cover changes in one step as different classes.

We followed a supervised classification based on a minimal set of informative transition classes: forest, non-forest, water, and cloud/shade (no data). The ‘forest’ class included old growth forest, secondary and degraded forest, as well as forest plantations. The ‘non-forest’ class depicted areas without tree cover and included natural non-forested areas such as wetlands and coastal plains. The use of baseline change classes should be useful for large-scale mapping of cover change in complex and heterogeneous areas, owing to the difficulties in discriminating some vegetation types based solely on spectral information [30]–[32]. The vegetation types that occur in Mexico are extremely varied as a result of its diverse climate and physiography. Mexico has a high diversity of ecosystems as a result of the transitional biogeographic zone running through the country and forming a bridge between the Nearctic (North American) and Neotropical (Central and South American) realms [25], [26]. This heterogeneity, linked to a long disturbance history in the region, poses difficulties to mapping the different vegetation types [33]. Even intermediate resolution remotely sensed data cannot accurately discriminate between certain forest categories [30].

We trained multi-date composites for all observed combinations of these change classes, *i.e.* forest loss, forest gain/recovery, and forest stability/maintenance. ‘Forest loss’ was defined as any change in the density and structure of forest vegetation that reduces tree cover, and is detectable as an alteration in the spectral response of the land surface through remote sensing. Forest loss could be caused by deliberate planned deforestation, or it could be the result of disturbances such as forest fires, hurricanes, floods or land slides. The latter effects may be considered as an intrinsic part of the natural disturbance regime of the area, however natural disturbance frequently acts in synergy with human activities thus leading to permanent deforestation [34]. In addition, disturbances such as forest fires are often linked to agricultural activities. This leads to a challenge in decoupling anthropogenic from natural change regimes. The methodology was therefore designed to provide clear evidence of any change in cover rather than documenting more subtle forest disturbance or changes in successional stage. As images were taken during the dry season, spectral changes due to phenological differences of vegetation were expected to be lower than changes due to land cover transformation. ‘Forest gain/recovery’ was defined as a reversion of open land back to forest, and may include both forest plantations and passive revegetation. ‘Forest stability/maintenance’ referred to no detectable changes in forest cover.

Training sites were identified through ground truthed data obtained through site visits, and visual interpretation of the images based on aerial photography and high resolution imagery from Google Earth. The process of classification was iterative. After a set of signatures representing all observed combinations of basic classes was created, the classification was run and the resulting thematic image was inspected for errors with reference to both dates of the satellite image pair. Errors were corrected by editing the training sites of existing signatures and/or creating additional signatures. This iterative process continued until visual inspection of the classification revealed no further obvious errors. Images were classified using the Sequential Maximum A-Posteriori algorithm [35].

Each two separate multi-date classification (*i.e.* 1990–2000 and 2000–2006 composites) corresponding to the same row and path, were unified to produce a single matrix. Where this led to contradictions (*e.g.* forest1990-nonforest2000, forest2000-nonforest2006) the results were investigated by eye and corrected. Unified classifications were finally joined to produce a three-date (*i.e.* 1990–2000–2006) continuous mosaic of forest cover and deforestation (available as supplementary information - **File S1**). The number of isolated pixels was reduced using a 4×4 modal filter, the results aggregated, and groups with less than 6 pixels eliminated.

### Accuracy Assessment

An accuracy assessment of the classification results was carried out and a confusion matrix and associated Kappa coefficients of agreement [36] generated. We used a random sample of 13,892 points derived from high resolution imagery in Google Earth with dates established as 2005, 2006 and 2007. We also used 295 field points from Chiapas, georeferenced in areas that had undergone deforestation during the study period. We considered a separation distance of at least 2 km between points.

### Forest Cover Change Assessment

In order to analyse forest cover dynamics, we used the resulting mosaic to calculate region wide and divisional forest cover and deforestation rates based on change intervals of 10 years (ca. 1990–ca. 2000), and 6 years (ca. 2000–ca. 2006).

Maps of state and municipality boundaries, ecological regions as proposed by Olson et al. [37] (**Figure 1a**), and federal protected areas (**Figure 1b**), were overlain on the resulting mosaic. Area statistics for each combination of classes were calculated. Total areas of forest loss and forest gain were calculated by summing the total area of pixels classified as forest loss and forest gain, respectively. The difference between forest loss and forest gain was defined as net change. Ecoregions that consisted of wetlands or with most of the area outside the study region were discarded from the analysis. Unclassified pixels (*i.e.* cloud/shadow and water) were not included in the estimates of total land area.

Average annual rates of forest loss, forest gain and net change, were calculated with the formula proposed by the Food and Agriculture Organization [38]:


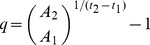


where *A*_1_ and *A*_2_ are the forest cover at time *t*_1_ and *t*_2_, respectively. Hotspots of deforestation were identified for more detailed investigation where more than 10% of the total area of the municipal district was found to have experienced forest loss during the period between 1990 and 2006.

In addition to calculating single point estimates of change for each time period, we used linear mixed-effects models in order to aggregate the trajectories in the rates of change from the 1990s to 2000s. At the municipal level mixed-effects models provide a powerful tool for the analysis of data that consist in repeated measurements on the same statistical units. In the context of this study the statistical units are municipalities. A single common (average) intercept and slope is fitted with the deviations for each municipality being treated as random effects. Details on mixed-effects modelling can be found in Zuur et al. [39]. The use of time as an explanatory variable led to a test of the EKC hypothesis, as the EKC would predict a reduction in the rate of forest loss and an increase in the rate of forest gain. Physical and socio-economic variables were also used as covariates in order to investigate other elements that could be associated with a change in trajectory. A series of models of varying complexity were built using the R package ‘nlme’ [40]. We adopted a top-down approach for model evaluation and selection following Zuur et al. [39]. Models were evaluated using information criteria (AIC). The following variables were used: (1) ‘mean elevation’, which was calculated from the WorldClim site digital elevation model (Available: http://www.worldclim.org/current. Accessed 2011 Jun 15); (2) ‘population density’, which was obtained from census-based population data for Mexican municipalities at multiple time points: *i.e.* the 1990, 2000 and 2005 population census by the Instituto Nacional de Estadística y Geografía [41]–[43]; and (3) ‘income per capita’ in 2000 and 2005, which was obtained from the United Nations Development Program (PNUD-México) [44]. Per capita income estimates rely on the statistical procedure by Elbers, Lanjouw and Lanjouw [45]. It consists in combining data from detailed household sample surveys that include reasonable measures of income (*i.e.* the national surveys on household income and expenditure by the Instituto Nacional de Estadística y Geografía), together with the comprehensive coverage of population census (*i.e.* the population census by the Instituto Nacional de Estadística y Geografía). A series of variables in a household dataset are identified (*e.g.* welfare variables, housing characteristics, education, labour situation) that fulfill two conditions: they are correlated with the data on income while at the same time they are surveyed in a population census from the same date. These variables are used for fitting predictive models of income, and fitted models are finally applied to the development of income measures for the entire censused population [44]. This variable therefore will be highly correlated by definition with most other measured socio-economic indices, leading to redundancy if these indices were included in a more complex statistical model. We therefore limited the models to include only population density and income as potentially orthogonal socio-economic variables. Although the two variables are significantly correlated (p<0.001, n = 342) the relationship is non linear and relatively weak (Spearman’s *rho* = 0.19, Pearson’s *r* = 0.37, adjusted R^2^ = 0.14) allowing for a large amount of uncorrelated variability.

Results from three discrete steps along a temporal sequence are insufficient to allow the shape of the trajectory to be modelled in detail. Therefore, as an extension to the temporal analysis of change trajectories, we investigated the spatial pattern in the proportion of remaining forest cover at the municipal level. This “space for time” substitution allowed an analysis in terms of the form and shape of the relationship of the proportion of remaining forest in each of the study periods with any of the socio-economic variables that had been found to be significantly associated with change as a result of the linear mixed effects modelling.

In the space for time analysis the proportion of forest remaining (rather than deforestation rates) was used as the response variable under the assumption that this variable integrates long periods of historical deforestation and land use change. It may thus be considered to be a better indicator of the overall long term effect of the population on forest cover than an instantaneous rate of change. Proportions are bounded between zero and one leading to asymmetric error distributions [46], [47]. This makes the beta distribution an appropriate model for residual error. Spline based models that allow relationships to take flexible forms are required in order to detect the curvilinear pattern predicted by Kuznets that would be obscured by linear modelling or correlation analysis. These considerations led to the use of generalised additive models (GAMs) which fitted cubic splines with beta distributed errors.

Beta GAMs have been implemented in R as part of the ‘gamlss’ package [47]. Beta GAMs are semi-parametric regression models that allow for non linear impacts of the explanatory variables to be modelled. They are part of the generalized additive models family. Details on GAMLSS modelling can be found in Rigby & Stasinopoulos [47] and Stasinopoulos & Rigby [48].

This modelling approach was aimed at determining the shape of underlying relationships that would otherwise be obscured by the large amount of inherent noise in a data set that integrates results over an extremely heterogeneous region. Parametric inference regarding the form of a relationship modelled as a spline is not possible. Therefore in order to test the statistical significance of the shape of the curve we used a bootstrap. One thousand random samples of municipalities were drawn with replacement from the data set. The models were refitted to these bootstrap samples and the results scored for compatibility with the form of the Kuznets curve in order to produce a conditional probability based significance test that took into account the potential effect on the shape of the relationship of a few influential points.

## Results

### Accuracy Assessment

Confusion matrices and Kappa estimates are shown in **Table 1**. Prior to the analysis, validation data were classified into forest types according to the ecoregions defined by Olson et al. [37]. Deforestation validation sites were obtained for all ecoregions. The Kappa index of agreement was 0.88, and overall agreement was 92.2 percent. The estimated Kappa is below 0.90 for tropical dry forests and pine-oak forests, and over 0.90 for tropical moist forests and montane cloud forests. Deforestation was generally accurately classified, although there were high omission rates for dry forest and pine-oak forest. Accuracy of cover change analysis using Landsat imagery will inevitably be lower in areas of dry and open forests than moist closed forests as the forest spectral signature will be less distinct from pasture and agricultural areas.

**Table 1:** Confusion matrices and estimated Kappa for forest classes and forest loss.

	Reference data^a^								
Classified data^a^	TDF	TMF	POF	MCF	FL	NF	Total	Error of Commission (%)	Estimated Kappa
TDF	2488	0	0	0	19	297	2804	11.27	0.86
TMF	0	2644	0	0	0	177	2821	6.27	0.92
POF	0	0	695	0	4	87	786	11.58	0.88
MCF	0	0	0	549	0	30	579	5.18	0.95
FL	0	0	0	0	272	0	272	0	1
NF	127	179	122	63	0	6434	6925	7.09	0.86
Total	2615	2823	817	612	295	7025	14187		
Error of Omission (%)	4.86	6.34	14.93	10.29	7.79	8.41			

a Abbreviations for forest classes and forest loss: Tropical dry forest ecoregions (TDF); tropical moist forest ecoregions (TMF); pine-oak forest ecoregions (POF); montane cloud forest ecoregions (MCF); forest loss (FL); no-forest (NF).

### Forest Cover Changes at the State Level

**Figure 2:**
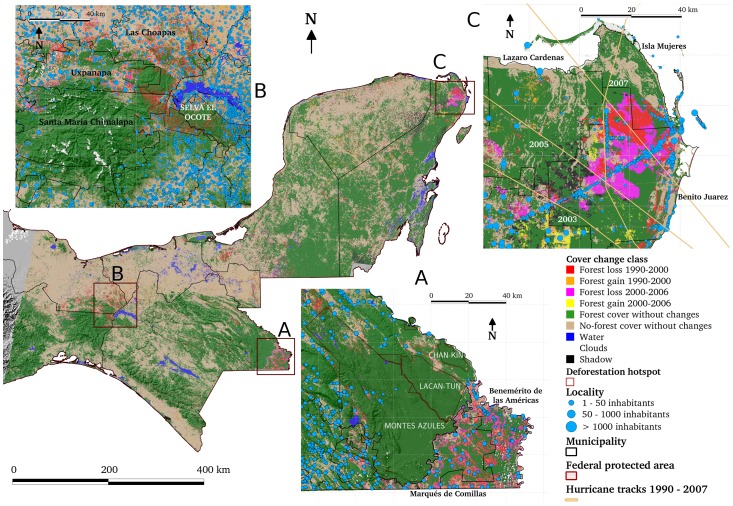
Cover change classification and deforestation hotspots in Southern Mexico. Hotspots were identified where more than 10% of the total municipal land area was losing forest cover during the entire study period (see Figure 3). A) Lacandon forest hotspot; B) Northern Chimalapas hotspot; and C) Benito Juárez-Isla Mujeres hotspot.

**Figure 3:**
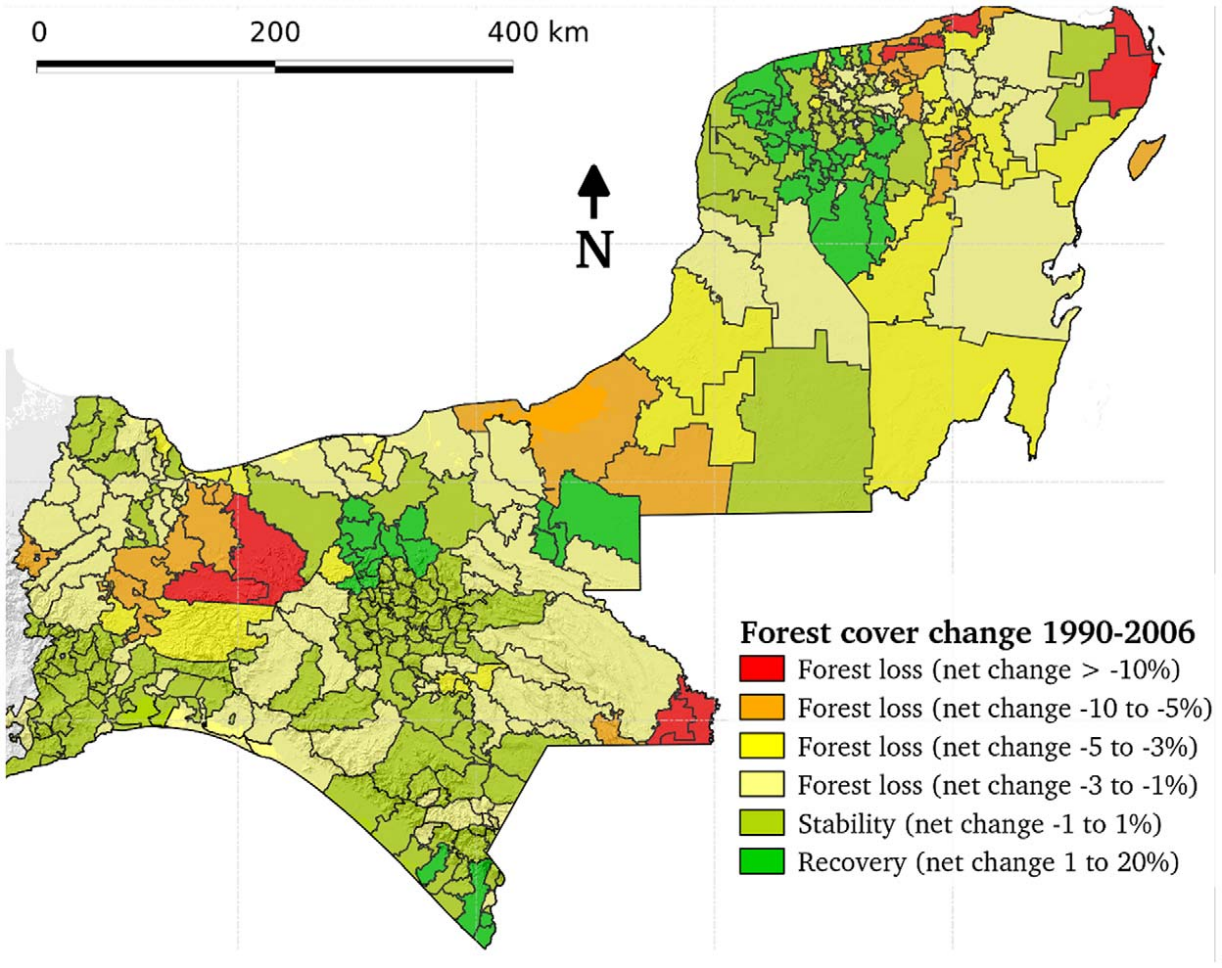
Net cover change between 1990 and 2006 calculated as a percentage of total land area in each municipality. Deforestation hotspots (depicted in red) were identified based on municipalities with more than 10% of the total land area that had changed from forest to non forest during this period.

**Figure 2** displays the spatial distribution of changes in Southern Mexico and the location of deforestation hotspots. These were identified as municipal districts in which over 10% of the land area had lost forest cover during the period 1990–2006 (see **Figure 3**). This definition took into account the wide range of differences in municipal areas and initial forest cover. **Table 2** shows a detailed picture of changes within states and the entire region. Tabasco, Yucatán and the area of Veracruz that falls within the study region showed the lowest percentages of remaining forest at the beginning of the study. During the study period, Tabasco experienced net reforestation (particularly within the Petén-Veracruz Moist Forest) and substantial forest recovery took place in Yucatán (particularly within the Yucatán Dry Forest). In Veracruz, deforestation during the 1990s was mostly concentrated in the foothills of the uplands (particularly within Sierra De Los Tuxtlas). The rate slowed down during the 2000s. Quintana Roo, Campeche, Chiapas and the area of Oaxaca that falls within the study area remain extensively forested and have also undergone a downward trajectory in net deforestation from the 1990s to 2000s.

**Table 2:** Single point estimates of change in forest area for each time period by state and overall Southern Mexican region.

State name	Area (ha)	Forest cover area in ha (% forest cover area)			Total forest loss in ha(% annual rate)		Total net change in ha(% annual rate)	
		1990	2000	2006	1990–2000	2000–2006	1990–2000	2000–2006
Tabasco	2,293,546	320,872 (14.0)	340,231 (14.8)	322,668 (14.1)	−52,257 (−1.76)	−17,572 (−0.88)	19,360 (0.59)	−17,563 (−0.88)
Veracruz	2,146,550	439,563 (20.5)	337,373 (15.7)	327,032 (15.2)	−108,796 (−2.80)	−10,975 (−0.55)	−102,191 (−2.61)	−10.341 (−0.52)
Chiapas	7,187,712	3,702,056 (51.5)	3,620,110 (50.4)	3,579,399 (49.8)	−135,711 (−0.37)	−40,727 (−0.19)	−81,946 (−0.22)	−40,711 (−0.19)
Yucatán	3,725,287	1,348,997 (36.2)	1,340,390 (36.0)	1,306,575 (35.1)	−98,472 (−0.76)	−38,328 (−0.48)	−8607 (−0.06)	−33,814 (−0.42)
Quintana Roo	4,675,433	3,321,905 (71.0)	3,190,321 (68.2)	3,137,660 (67.1)	−156,640 (−0.48)	−75,793 (−0.40)	−131,584 (−0.40)	−52,661 (−0.28)
Oaxaca	2,537,552	1,746,372 (68.8)	1,713,246 (67.5)	1,697,387 (66.9)	−33,312 (−0.19)	−15,864 (−0.15)	−33,126 (−0.19)	−15,859 (−0.15)
Campeche	4,984,545	2,983,837 (59.9)	2,874,919 (57.7)	2,819,381 (56.6)	−122,285 (−0.42)	−60,067 (−0.35)	−108,918 (−0.37)	−55,538 (−0.32)
Overall region	27,550,626	13,863,602 (50.3)	13,416,590 (48.7)	13,190,102 (47.9)	−707,474 (−0.52)	−259,327 (−0.32)	−447,013 (−0.33)	−226,488 (−0.28)

### Forest Cover Changes within Ecological Regions

**Table 3** summarises changes by ecoregion. Tropical dry forests showed the lowest percentages of remaining forest at the beginning of the study. Forest cover extent remained higher in the Yucatan Dry Forest ecoregion. Deforestation during the study period mainly occurred in small patches and there was little evidence of extensive contiguous areas of forest loss (**Figure 2**). However substantial deforestation has continued threatening dry forests in Yucatán particularly around Cancun. Low deforestation rates within most drylands together with a substantial increase in forest cover, contrast with the advanced state of conversion and disruption of these dry forest landscapes.

**Table 3:** Single point estimates of change by ecoregion in Southern Mexico.

Ecoregion^a^	Area (ha)	Forest cover area in ha (% forest cover area)			Total forest loss in ha(% annual rate)		Total net change in ha(% annual rate)	
		1990	2000	2006	1990–2000	2000–2006	1990–2000	2000–2006
CADF	321,806	24,713 (7.7)	34,276 (10.6)	32,171 (10.0)	−3030 (−1.30)	−2105 (−1.05)	9563 (3.33)	−2105 (−1.05)
CHDDF	1,247,565	419,208 (33.6)	412,812 (33.1)	411,401 (33.0)	−6447 (−0.15)	−1411 (−0.06)	−6396 (−0.15)	−1411 (−0.06)
YDF	4,905,012	2,081,671 (42.4)	2,060,732 (42.0)	2,024,533 (41.3)	−125,037 (−0.62)	−45,229 (−0.37)	−20,939 (−0.10)	−36,199 (−0.29)
All tropical dry	6,474,383	2,525,592 (39.0)	2,507,821 (38.7)	2,468,105 (38.1)	−134,513 (−0.55)	−48,745 (−0.33)	−17,772 (−0.07)	−39,715 (−0.27)
PVMF	6,619,008	2,790,410 (42.2)	2,598,606 (39.3)	2,535,676 (38.3)	−278,131 (−1.04)	−63,308 (−0.41)	−191,804 (−0.71)	−62,930 (−0.41)
ST	375,913	60,077 (16.0)	55,733 (14.8)	55,759 (14.8)	−5696 (−0.99)	−308 (−0.09)	−4344 (−0.75)	26 (0.01)
YMF	6,705,716	4,831,974 (72.1)	4,648,895 (69.3)	4,555,664 (67.9)	−204,554 (−0.43)	−115,923 (−0.42)	−183,079 (−0.39)	−93,231 (−0.34)
All tropical moist	13,700,637	7,682,462 (56.1)	7,303,234 (53.3)	7,147,098 (52.2)	−488,382 (−0.65)	−179,539 (−0.41)	−379,228 (−0.50)	−156,136 (−0.36)
SMMF	538,955	386,146 (71.6)	381,129 (70.7)	379,494 (70.4)	−8495 (−0.22)	−1635 (−0.07)	−5017 (−0.13)	−1635 (−0.07)
POF	1,590,606	933,379 (58.7)	918,788 (57.8)	916,279 (57.6)	−15,037 (−0.16)	−2508 (−0.05)	−14,591 (−0.16)	−2508 (−0.05)
CHMF	556,316	381,061(68.5)	375,553 (67.5)	375,220 (67.4)	−6104 (−0.16)	−333 (−0.01)	−5508 (−0.15)	−333 (−0.01)
CMF	206,652	157,248 (76.1)	156,676 (75.8)	156,511 (75.7)	−604 (−0.04)	−165 (−0.02)	−572 (−0.04)	−165 (−0.02)
Mountain ecoregion	2,892,530	1,857,834 (64.2)	1,832,146 (63.3)	1,827,504 (63.2)	−30,240 (−0.16)	−4642 (−0.04)	−25,689 (−0.14)	−4642 (−0.04)

a Abbreviations for ecoregions: Central American Dry Forest (CADF); Chiapas Depression Dry Forest (CHDDF); Yucatan Dry Forest (YDF); Petén-Veracruz Moist Forest (PVMF); Sierra de Los Tuxtlas (ST); Yucatán Moist Forest (YMF); Sierra Madre de Chiapas Moist Forest (SMMF); Central American Pine-Oak Forest (POF); Chiapas Montane Forest (CHMF); Chimalapas Montane Forest (CMF).

Regional-scale losses occurred within all the tropical moist forest ecoregions, and revegetation was comparatively modest. Deforestation affected most of the ecoregions (**Figure 2**). However deforestation rates followed a downward trajectory from the 1990s to 2000s. Also, there were extensively forested areas within which forest cover has remained stable during the study period (**Figure 2**). The limited extent of remaining forest in Sierra De Los Tuxtlas at the beginning of the study, contrasts with the other tropical moist forest ecoregions. The estimated percent of remaining forest in 1990 coincides with that of the more detailed case study of Dirzo & García [49]. Deforestation continued threatening this ecoregion during the 1990s; however by the 2000s deforestation had slowed.

Montane ecoregions remain extensively forested. During the 1990s deforestation was comparatively modest, and slowed by the 2000s. The pine-oak forests of Central America extend along two different mountainous subregions of Chiapas: the Highlands and the Sierra Madre, which run west-east through the central and eastern portions of the state, respectively (see **Figure 1a**). The methodology used in this study detected a clear focal area of deforestation around the community of Betania, between San Cristóbal de Las Casas and Teopisca. Deforestation in this area was intense during the 1990s, but it was significantly decreased during the 2000s. Many other localised areas of deforestation are noticeable within this ecosystem, although no montane municipality was registered as a hotspot under our definition. Relatively localised nuclei of deforestation at the level of an individual community are often linked to social issues connected with religious and political conflicts in the region.

### Deforestation Hotspots

Two hotspots of deforestation were detected within the Petén-Veracruz Moist Forest ecoregion (**Figure 2** **and** **3**). The lowland forests of the Lacandon subregion make up the largest continuous extent of moist tropical forest in Southern Mexico. Land use change threatens the edges of the reserves of Montes Azules and LacanTun and the matrix of fragmented and disturbed forest in the surrounding areas: Benemérito de las Américas and Marqués de Comillas districts. In these municipalities the study suggests that 58,794 ha of an original area of around 158,000 ha of forest were lost from 1990 to 2006. In the Northern Chimalapas hotspot, 91,388 ha of an area of around 804,000 ha of forest were lost from 1990 to 2006. High rates of deforestation were also detected in the reserve of El Ocote and surrounding areas. Another deforestation hotspot was detected to the north portion of Quintana Roo (**Figure 2** **and** **3**), within the Yucatán Moist Forest ecoregion. In this area, 76,576 ha of an original area of around 281,800 ha of forest were lost from 1990 to 2006. Hotspots of deforestation thus appear to be areas of recent colonization where the initial steep downward trajectory of the EKC would be expected to be most pronounced.

### Forest Cover Changes within Large Forest Reserves

Aggregated results by large forest reserves are summarised in **Table 4**. Deforestation during the last two decades has been comparatively modest, except for Los Tuxtlas Biosphere Reserve. Forest cover has remained relatively stable during the 2000s. However, within the reserves of La Sepultura and La Frailescana, deforestation continues to reduce forest cover.

**Table 4:** Single point estimates of change by large forest reserves in Southern Mexico.

Reserve name	Area (ha)	Ecoregion^a^	Forest cover area in ha (% forest cover area)			Total forest loss in ha (% annual rate)		Total net change in ha (% annual rate)	
			1990	2000	2006	1990–2000	2000–2006	1990–2000	2000–2006
Cañón Sumidero^b^	19.501	CHDDF	10,668 (54.7)	10,594 (54.3)	10,593 (54.3)	−74 (−0.07)	−1 (−0.00)	−74 (−0.07)	−1 (−0.00)
Calakmul^c^	723,591	YMF	663,626 (91.7)	660,592 (91.3)	660,318 (91.3)	−3118 (−0.05)	−275 (−0.01)	−3034 (−0.05)	−275 (−0.01)
Balan Kaax^d^	128,789	YMF	117,017 (90.9)	116,506 (90.5)	116,262 (90.3)	−850 (−0.07)	−256 (−0.04)	−512 (−0.04)	−244 (−0.03)
Sian Kaan^c^	286,735	YMF	149,095 (52.0)	149,049 (52.0)	148,959 (52.0)	−315 (−0.02)	−191 (−0.02)	−46 (−0.00)	−90 (−0.01)
Los Tuxtlas^c^	153,920	ST	49,508 (32.2)	46,165 (30.0)	46,469 (30.2)	−4441 (−0.94)	−26 (−0.01)	−3343 (−0.70)	303 (0.11)
Montes Azules^c^	323,280	PVMF	303,163 (93.8)	298,520 (92.3)	297,162 (91.9)	−4743 (−0.16)	−1357 (−0.08)	−4643 (−0.15)	−1357 (−0.08)
Lacan-tun^c^	61,822	PVMF	60,459 (97.8)	60,080 (97.2)	59,964 (97.0)	−381 (−0.06)	−115 (−0.03)	−380 (−0.06)	−115 (−0.03)
Chan-kin^d^	12,178	PVMF	12,071 (99.1)	12,005 (98.6)	12,005 (98.6)	−66 (−0.05)	−1 (−0.00)	−66 (−0.05)	−1 (−0.00)
Selva El Ocote^c^	101,007	PVMF	69,780 (69.1)	67,903 (67.2)	67,824 (67.1)	−1877 (−0.27)	−79 (−0.02)	−1877 (−0.27)	−79 (−0.02)
La Sepultura^c^	166,281	POF	114,952 (69.1)	113,510 (68.3)	112,552 (67.7)	−1445 (−0.13)	−974 (−0.14)	−1442 (−0.13)	−958 (−0.14)
El Triunfo^c^	118,236	SMMF	94,159 (79.6)	92,271 (78.0)	92,040 (77.8)	−1940 (−0.21)	−231 (−0.04)	−1888 (−0.20)	−231 (−0.04)
La Frailescana^e^	154,126	POF-SMMF	117,826 (76.4)	115,808 (75.1)	114,780 (74.5)	−2322 (−0.20)	−1028 (−0.15)	−2018 (−0.17)	−1028 (−0.15)
Mozotal-Tacana^c^	199,350	SMMF-POF	141,372 (70.9)	139,579 (70.0)	139,437 (69.9)	−2329 (−0.17)	−142 (−0.02)	−1792 (−0.13)	−142 (−0.02)
Pico Loro Paxtal^f^	60,891	POF	45,342 (74.5)	44,718 (73.4)	44,681 (73.4)	−623 (−0.14)	−37 (−0.01)	−623 (−0.14)	−37 (−0.01)

a Chiapas Depression Dry Forest (CHDDF); Petén-Veracruz Moist Forest (PVMF); Sierra de Los Tuxtlas (ST); Yucatán Moist Forest (YMF); Sierra Madre de Chiapas Moist Forest (SMMF); Central American Pine-Oak Forest (POF); Chiapas Montane Forest (CHMF).

b National Park;

c Biosphere Reserve;

d Area for Protection of Flora and Fauna;

e Area for Protection of Natural Resources;

f State Reserve.

The effect of protected areas on deforestation can be inferred by contrasting the statistics on each of the protected areas with those on each of the surrounding ecoregions in which they are located (*i.e.* **Table 4** vs. **Table 3**): (1) The proportion of forested area is consistently higher within protected areas than within their surrounding ecoregions. The biosphere reserve of Sian Kaan is the exception; however this is an assembly of ecosystems (*i.e.* forests, wetlands, coastal and marine). (2) Protected areas in the lowland tropics show significantly lower rates of deforestation than their surrounding ecoregions, except for Los Tuxtlas Biosphere Reserve. Most deforestation in Sierra De Los Tuxtlas was localised within Los Tuxtlas reserve. (3) Deforestation rates within and outside the reserves in mountainous subregions have been consistently low.

### Forest Transition

The cover change analysis presents a complex pattern of deforestation in the region. Statistically significant patterns must be placed in the context of heterogeneity resulting from unique histories of deforestation and cover change at a sub regional (municipal) level. The results from linear mixed-effects models on rates of forest loss and forest gain are shown in **Table 5** and **Table 6**, respectively. A random intercept model was selected by AIC based model comparison as the random component for both cases. Significant interactions between variables were not detected; therefore interaction terms were dropped from models.

**Table 5:** Results from linear mixed-effects models on rate of forest loss.

A					
Fixed effects: rate of forest loss ∼ mean elevation + time + income per capita + population density(AIC value = 2200)					
	Value	Std. Error	DF	t-value	p-value
Intercept	1.0807	0.1539	340	7.0184	0.0000
Mean elevation	−0.0006	0.0001	340	−6.3430	0.0000
Time	−0.3240	0.0974	323	−3.3253	0.0010
Income per capita	0.0001	0.0000	323	2.9992	0.0029
Population density	0.0011	0.0005	323	2.3181	0.0211
**B**					
**Fixed effects: rate of forest loss ∼ mean elevation + time + population density** **(AIC value = 2208)**					
	**Value**	**Std. Error**	**DF**	**t-value**	**p-value**
Intercept	1.2199	0.1482	340	8.2297	0.0000
Mean elevation	−0.0008	0.0001	340	−7.8899	0.0000
Time	−0.1833	0.0865	324	−2.1191	0.0348
Population density	0.0017	0.0005	324	3.6614	0.0003

A) Fixed effects including all the variables. B) Fixed effects excluding income per capita. According to AIC values, the random intercept model was the optimal structure of the random component.

**Table 6:** Results from linear mixed-effects models on rate of forest gain.

Fixed effects: rate of forest gain ∼ time(AIC value = 1911)					
	Value	Std. Error	DF	t-value	p-value
Intercept	1.1289	0.1174	341	9.6162	0.0000
Time	−0.5432	0.0742	341	−7.3208	0.0000

According to AIC values, the random intercept model was the optimal structure of the random component.

The optimal model for predicting rates of forest loss included all the variables analysed (**Table 5a**). Rate of forest loss decreased from the 1990s to 2000s. Annual rates averaged 0.85 (median = 0.32) for the 1990s, and 0.67 (median = 0.13) for the 2000s. Rate of forest loss was found to be negatively related to elevation. Mean elevation is interpreted as a surrogate of topographic complexity thus representing a physical constraint that imposes limitations on land use. The results therefore indicate that the likelihood of deforestation will be lower as the terrain becomes steeper and more inaccessible. Population density and income per capita were also significant predictors of forest loss. Social data for municipalities matched to the foregoing rates of forest loss come from the 1990 and 2000 population census, and from the 2000 and 2005 data on income per capita. Population densities differ markedly among municipalities and recent deforestation rates are positively related to local human population density. A strong correlation between population density and tropical deforestation has been demonstrated by recent studies [50]–[52]. However this relationship must be interpreted with great care as the overall shape may not be linear. Collinearity between predictor variables may also confound interpretation. The statistical significance of population density as a predictor variable of forest loss was markedly increased when income was excluded in the statistical model (see **Table 5b**). This was due to correlation between the two social factors. A space for time analysis that used models of a non linear form helped to clarify the effects of these factors.

For the model on rates of forest gain, time was the only significant variable among all the variables analysed. Unexpectedly, rate of forest gain decreased from the 1990s to 2000s (**Table 6**). Annual rates averaged 0.58 for the 1990s, and 0.04 for the 2000s. Because the second study period was shorter than a decade there may have been some underestimation of the extent of forest gain during the 2000s. However forest gain has been not a common process during the study period (median 1990–2000 = 0.02; median 2000–2006 = 0.00). Only a few municipalities within largely deforested subregions have experienced a significant forest recovery (see **Figure 3**).

**Figure 4:**
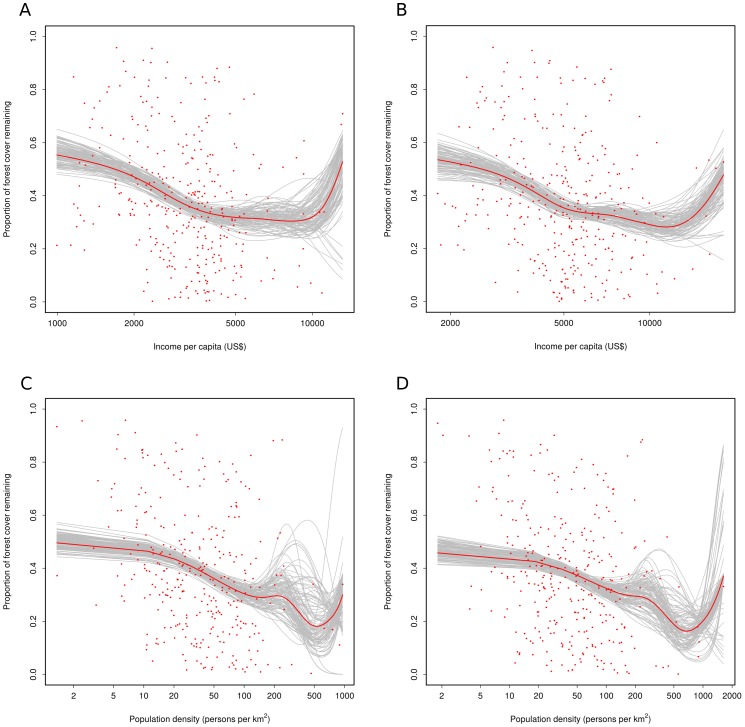
Beta GAM modelling on the relationship between the proportion of remaining forest cover at the municipality level and socio-economic factors. A) Relationship between the proportion of forest cover remaining in 2000 and income per capita in 2000 (Deviance explained = 14.5%); B) Relationship between the proportion of forest cover remaining in 2006 and income per capita in 2005 (Deviance explained = 11.9%); C) Relationship between the proportion of forest cover remaining in 1990 and population density in 1990 (Deviance explained = 22.0%); and D) Relationship between the proportion of forest cover remaining in 2006 and population density in 2005 (Deviance explained = 12.8%). The bootstrapped cases and the fitted models with all the data are shown in grey and red lines, respectively. Figure 4a and 4b provide support for Kuznets’ hypothesis. Although the scatter around the trend is substantial leading to a small amount of explained deviance (<15%) the underlying relationships are a close match to that followed by an EKC. Economic development has been comparatively rapid in the region between 2000 and 2005 with approximately 5% growth in per capita income annually, leading to a change in spread along the abscissa. The figures show the highly skewed nature of income distribution, with a few municipalities that rely largely on tourism for income having per capita incomes over US$ 10,000 while most rural communities lie below US$ 5000 in both time periods. Figures 4c and 4d also display the same intrinsic variability. The model of the underlying trend shows a decline in forest cover in rural areas as population density increases up to a threshold of 100 people per km^2^. The trend is approximately linear on a log scale, so the gradient decreases along this part of the trajectory when the curve is plotted on a linear scale. The loss in cover slows and is ultimately reversed, even on a log scale, at a population density of around 100 persons per km^2^. Forest cover may decrease once again at levels of population density that are typical of densely urbanised areas, followed by an increase in the largest, long established cities (perhaps as parks and gardens are established). There are very few data points available to provide support for this part of the curve, so the bootstrapped curves take varying forms beyond the 100 person per km^2^ cutoff level. The shape of the right hand side of the curve is thus not established through these data.

The results from beta GAM modelling are shown in **Figure 4**. A large amount of scatter around any underlying trend is to be expected in an analysis that uses a space for time substitution. Each municipal district has a unique combination of environmental conditions together with its own history of colonization and development. However the shape of the underlying relationship follows the form predicted by Kuznets. The bootstrap analysis confirmed that the trend was robust given the data obtained. Although some bootstrap samples led to fitted models with alternate shapes, over 95% of 1000 plotted bootstrapped curves displayed a shape that included a reduction in slope on the log scale, usually followed by a slight upturn at very high income levels. A similar result was obtained through bootstrapping the relationship between forest cover and population density. Over 95% of the curves had shape that represented a flattening of the decline in forest cover in relation to population density on a log scale. Without log transformation both patterns were visually accentuated with the slope of the curve declining markedly in response to increases in either population density or income.

## Discussion

### Forest Cover Changes in Southern Mexico

#### Tropical dry forests

The extent of dry forests in Southern Mexico was greatly reduced before the 1990s, mainly as a consequence of agricultural and cattle farming development. Other dry forests in Mexico have also been extensively deforested [53]. The current pattern of usage in the Chiapas Depression Dry Forest has its origins in the haciendas established in the region in the seventeenth century. Land use intensified throughout the twentieth century as a result of cattle production and sugar cane cultivation [54]. Human settlement and monoculture forestry in the Central American Dry Forest, as well as henequen plantation in the Yucatán Dry Forest all contributed to dry forest loss [55], [56]. The recent stabilization of forest cover in the Central Depression of Chiapas and the net forest recovery in the coastal drylands of the state, suggest that a cessation, or at least a significant fall, in agricultural and cattle production expansion, has taken place in these regions. High resolution imagery from Google Earth, suggest that most of the forest gain that took place in the coastal drylands of Chiapas was driven by plantation forestry, particularly oil palm. Oil palm plantations have been stimulated by government subsidies that have been promoting production on abandoned land that had previously been deforested by cattle raising and other activities that are no longer profitable in this region [57], [58]. According to the author, a total of 16,298 ha of oil palm were planted in Chiapas from 1996 to 2001. It is debatable whether oil palm plantations should be considered to represent reforestation. In contrast, the total area planted with oil palm in Mexico decreased during the 2000. In Chiapas approximately 9,000 ha were lost from 2001 to 2004 mainly as a consequence of forest fires and floods, and many plantations were abandoned due to falling prices [58]. This fact may explain the shift from net reforestation to net deforestation that was detected in this ecoregion from the 1990s to 2000s. Although net deforestation has continued threatening dry forests in Yucatán, substantial secondary forest regrowth has taken place from henequen fields that have been abandoned due to falling prices and the decrease in world demand [55]. According to the authors, extensive forest clearings have continued to occur in this region since the late 1970s.

#### Tropical moist forests

Historical deforestation of moist forest has been driven mainly by cattle grazing and agriculture expansion [49], [59]–[61], affecting most of the lowland areas of Tabasco and Veracruz (see **Figure 2**). Our analysis shows that deforestation has continued to affect moist forests, but it has been displaced into new areas. Deforestation in the lowland forests of the Lacandon subregion was largely due to conversion of forest to agricultural use, mainly in the form of cattle pasture. This large scale conversion is the result of a deliberate policy of settlement, particularly in the area of Marqués de Comillas [62]. The area is key habitat for species requiring continuous expanses of undisturbed forest, including jaguars, tapirs and harpy eagles [63]–[66]. Most deforestation in Northern Chimalapas was driven by extensive uncontrolled forest fires linked to agricultural burning [67]. The high deforestation rates that were detected in the reserve of Selva El Ocote and surrounding areas, are in accordance with the results found by Flamenco-Sandoval et al. [68]. The building of a new motorway linking the states of Chiapas and Tabasco has also led to deforestation as improved communication allowed agricultural development and settlement. The Yucatán Moist Forest ecoregion is an important biological corridor that facilitates the movement of biota across the Yucatán peninsula and Central America [69]. The cause of forest loss in the hotspot of Quintana Roo is unclear. The area is hurricane prone, the most devastating event during the period analysed being hurricane Wilma in 2005 that passed directly over the area. Damage and fires were reported to have been very severe in portions of the Northeastern Yucatán peninsula [70].

In the central portion of Quintana Roo community forest management for timber has played an important role in forest cover maintenance [19]. The effectiveness of the Calakmul Biosphere Reserve in protecting forests has contributed largely to the maintenance of forest cover in Southeastern Campeche [69], [71]. Also, substantial forest recovery has taken place within the historically deforested lowlands of Tabasco. This forest recovery was mostly concentrated in Huimanguillo district, in Southwestern Tabasco (see **Figure 2**). A land use map of Huimanguillo [72] suggests that most of the forest gain coincides with areas of eucalyptus and citrus plantations, which is in accordance with Alejandro-Montiel et al. [73], who reported 7274 ha of eucalyptus planted in this area in 1996.

#### Montane forests

Annual cropping, mostly for maize and beans, is the land use whose expansion may have contributed to forest loss before the study period began [74]–[76]. The implementation of protected areas and the promotion of more sustainable economic opportunities appeared to be important drivers leading to forest cover maintenance in the last decades. For example, forest protection in the Sierra Madre of Chiapas relies on an extensive system of protected areas that includes the Biosphere Reserves of La Sepultura (decreed in 1995), El Triunfo (1990), and Volcán Tacaná (2003), the Area for the Protection of Natural Resources La Frailescana (1979), and the State Reserve Pico de Loro-Paxtal (2000). Important income-producing activities such as coffee plantation contribute to the maintenance of large areas with forest cover [77]. Many communities in montane areas that derive income from logging and forest management have maintained forest cover and restored density and commercial productivity in previously mismanaged forests [76], [78].

The limited amount of cover change that was detected in the Central American Pine-Oak Forest ecoregion contrasts with previous studies in the Highlands, which suggest intense ongoing disturbance and impact on remaining forests [74], [79]. The previous studies all adopted methodologies designed specifically to detect disturbance caused by small scale canopy opening rather than large scale cover change. This results in degradation rather than overall loss in cover. The method used by our study may thus be underestimating the extent of forest degradation.

### Forest Transition in Southern Mexico

Aggregated results suggest that the regional pattern of change during the study period was net deforestation (**Table 2**). However net deforestation has occurred at a lower rate compared with estimates from previous decades [80]–[83]. In general, Mexican deforestation estimates for the 1980s range from 0.8 to 2 percent annual rate [84]. A major pulse of deforestation took place within the study region from the 1960s to 1980s [22], [85]. This rapid change may have resulted in the widely held perception that deforestation in the region continues to accelerate. Differences between our results and other estimates of deforestation during the same period could be attributed to authors’ extrapolating previously observed trends beyond the evidence that they had available or studies that concentrated attention on deforestation hotspots [83]. Steininger et al. [86], for example, used a “wall-to-wall” remote sensing analysis to demonstrate that the rate of deforestation of the Bolivian Amazon was almost four times lower than that reported by the Food and Agriculture Organization in its 2000 assessment of global deforestation (also known as FRA 2000), which is based on a 10 percent sample remote sensing survey for tropical areas. The results of the current study support studies that have reported an overall reduction in deforestation rates at a regional scale [5], [22]. The results from linear mixed-effects models further confirm that the rate of forest loss has decreased from the 1990s to 2000s. In contrast, forest gain has been not been a common process, therefore transition has been extremely localised and has been the result of passive processes associated with reductions in the intensity of land use.

Linear mixed-effects models provide a direct interpretation of the strength of income and population density as drivers of deforestation. The significant correlation between income and population density suggests that population data may also act as a proxy for variables related to economic factors. Other multi-country and regional studies found that the correlation between population density and deforestation could not be detected when additional independent variables were added to the models, concluding that population acts as a proxy for other factors [87]. In this study, the beta GAM analyses show a more complex pattern that would be obscured by linear models. The functional form of the models suggest that the relationship between the remaining forest cover and socio-economic factors follows an inverted J-shaped curve, thus providing evidence to suggest that deforestation decline is reversed beyond certain population levels as per capita income becomes higher. Scatter around the trend was very high and a large proportion of the variability could not be explained by population density nor by economic factors. However a detectable underlying pattern is consistent with the EKC.

Municipalities and subregions with distinct settlement histories may serve as proxies for different stages along the forest transition, theorized to exhibit depletion of forest cover, eventually followed by a recovery. Hotspots areas, for example, are related to municipalities that show the most recent histories of human occupation and development within the study region. The large scale conversion in Benemérito de las Américas and Marqués de Comillas districts is the result of a deliberate policy of settlement in the Lacandon subregion [62], [88]. Deforestation in Las Choapas, Uxpanapa and Santa Maria Chimapala, is related to the expansion of human settlement into the unpopulated uplands of the Chimalapas region [67]. Finally, coastal municipalities in the Yucatan peninsula, especially Benito Juarez, have experienced high population growth during the last decades, which has been associated with the development of tourism activities [89]. In the other hand, Veracruz, Tabasco, Yucatan, and the Central Depression and the coastal plain of Chiapas, have a long history of continuous human occupation. Many municipalities in these regions are currently experiencing the stabilization or recovery of forest cover. Tabasco, for example, holds the highest population density (80.5 persons/km^2^) as compared with the rest of the states within the study region. Many municipalities in Veracruz and Tabasco are associated with a high population density since the beginning of the XX century, when a railway and highway were constructed connecting the city of Veracruz with other major cities in the region [49], [90]. Increased communication promoted the development of activities such as timber trading, sugar factories, tobacco plantations, and cattle ranching, which have had a marked impact on the forests of the region [90].

Research [91], [92] has found that although rural populations have the most direct impact on forests, urban populations influence demand for forest resources, market availability, human migration and other factors. In Southern Mexico the growth of many cities has been a consequence of inmigration from the rural hinterland, and the use of nearby forest may have intensified due to the extraction of elements such as charcoal, fuelwood, timber and ornamental plants for the urban market [21]. However economic growth may halt this trend as the urban population become reliant on gas and inter fuels for heating and begin to purchase food and other agricultural products that are imported from beyond the neighboring rural area.

The large amount of scatter and the low proportion of the variability explained by any model may be explained by other social and political processes constraining forest cover trends, such as land tenure and property rights. Patterns of land tenure in Southern Mexico play a critical role in shaping the mode of production. Different modes of production, in turn, are likely to be characterised by different demographic relationships through which the population-forest relationship is mediated [93]. The Highlands of Chiapas, for example, have an extremely high population density that can no longer be sustained through subsistence agriculture alone. In municipalities such as San Cristóbal de Las Casas, Chamula and Mitontic, the population density is above 200 people per km^2^. Yet a surprising amount of forest cover (>50%) still remains. This can be explained by cultural factors, including the retention of forest as a source of fuel, combined with the growth in off farm employment in the region. In contrast, the Central Depression of Chiapas has a much lower resident population and an entirely agricultural economy. Although this was once considered to be the wealthiest area of the state of Chiapas. However the rural economy based around agriculture has stagnated since 1990 as a result of low commodity prices and structural difficulties which have made commercial maize production noncompetitive. Incomes have fallen in relative terms in this area. In most municipalities of the central depression population density is below 100 people per km^2^, yet the area has reduced forest cover to below 10%. Laurance et al. [92] documented a similar pattern in Brazilian Amazonia, concluding that not all segments of the rural population are equally important drivers of deforestation. The results of this study also suggest that an institutional component leading to forest protection and a changing array of rural economic activities, play a significant role in dropping or stopping deforestation. Other authors [18], [19] have highlighted the positive effect of this institutional component on avoiding deforestation in Southern Mexico. Nevertheless the pattern of results of this study suggests that passive processes that have reduced the rate of deforestation are more important than active stimuli within the region.

Overall, Southern Mexico has retained extensive nature forests of some kind, although many are now degraded and of secondary origin. Approximately fifty percent of total land area was found to remain forested. This estimate is in broad agreement with results found by Toledo [82] and Masera et al. [94], although these authors have higher estimates of ongoing deforestation. Whether a transition will take place at such comparatively high levels of cover is debatable. Forest cover in Costa Rica, Puerto Rico and El Salvador declined to 30, 10 and 5 percent of total area, respectively, before a break point in forest cover decline took place [13], [95]–[97]. The time course of future trends in many subregions of Southern Mexico remains uncertain. In the lowland tropics, deforestation continues to occur at high rates in some focal areas, and intense deforestation pressures still persist in proximity to many forest reserves. In the Fraylesca region, surrounding La Sepultura and La Frailescana reserves, grassland continues to encroach into the forests of the reserves buffer zones [21]. In the Chimalapas region, human settlement is expanding from the lowlands into the undisturbed forests of the unpopulated uplands and threats the conservation of the forest [67]. Areas such as Marques de Comillas are unlikely to reach an equilibrium state of forest cover in the near future unless active steps are taken to deflect the current dynamic.

### Implications of the Change

If an incipient forest transition is occurring in Southern Mexico it could be accelerated if there were a broader recognition of its existence and its implications. There are opportunities to restore forest cover through actively stimulating afforestation and stimulating technological change toward more sustainable land use practices. Payments for ecosystem services (PES) are viable options when the pressure to deforest new lands is falling. PES projects can promote conservation while supporting the economic development of rural populations [98], [99]. Such initiatives are slowly expanding in tropical countries [100]–[103]. Forest management in Mexico has reached only a third of the area considered commercially viable. Some of the barriers to the spread of community-based forestry are economic and technical, related to inappropriate methods and a lack of capital and technical assistance [78]. Forms of adaptive and multifunctional land use such as mixed agroforestry systems should also be encouraged as an alternative to monoculture cropping and crop pasture.

### Conclusions

The study places cover change dynamics within a regional perspective. Results support the hypothesis of an incipient forest transition in Southern Mexico despite evidence of intense localised deforestation. The prevailing paradigm that deforestation continues to accelerate requires re-evaluation. The results confirm that the rate of forest loss has decreased from the 1990s to 2000s. Nevertheless forest gain has not been a common process. Transition has been extremely localised and has been the result of passive processes associated with reductions in the intensity of land use. The study suggests that the EKC could describe the relationship between remaining forest cover and socio-economic factors (*i.e.* income per capita and population density), although other factors are involved. Statistical modelling suggests that these relationships follow an inverted J-shaped curve, thus suggesting that deforestation decline is reversed beyond certain population levels as per capita income becomes higher. Although population pressure and economic factors are an important part of the explanation of tropical deforestation in Southern Mexico, other social and political processes (such as land tenure and the existence of institutions stimulating forest protection and technological change toward more sustainable land use practices) constrain forest cover trends. The incipient forest transition occurring in Southern Mexico provides opportunities to restore forest cover through actively stimulating afforestation and technological change toward more sustainable land use practices. However a broader recognition of its existence and implications is required in order to accelerate the transition to more sustainable landscapes. Efforts to reduce the rate, extent, location, or intensity of human-caused deforestation may become more successful in the future if they are aimed at accelerating an incipient trend. Strategies aimed at forest regrowth and tree planting may require further investment in order to succeed. Demographic pressure should receive less attention in policy measures directed at controlling deforestation.

## Supporting Information

File S1 **Raster file of the classification map.** Forest cover and change in Southeast Mexico: 1990–2000–2006. Cover classes are summarized into change classes using the following codes: 1 forest; 2 non-forest; 4 water; 0 cloud/shade (no data). Thus for example “forest 1990-nonforest 2000-nonforest 2006” was given a value of 122. Layer spatial reference system: +proj = longlat +ellps = WGS84+datum = WGS84+no_defs.
(ZIP)
